# Delayed cardiac tamponade after open heart surgery - is supplemental CT imaging reasonable?

**DOI:** 10.1186/1749-8090-8-158

**Published:** 2013-06-24

**Authors:** Bernhard Floerchinger, Daniele Camboni, Simon Schopka, Philipp Kolat, Michael Hilker, Christof Schmid

**Affiliations:** 1 Department Cardiothoracic Surgery, University Medical Center Regensburg 93053, Regensburg, Germany

**Keywords:** Cardiac tamponade, Computer tomography, Transthoracic echocardiography

## Abstract

**Background:**

Cardiac tamponade is a severe complication after open heart surgery. Diagnostic imaging is challenging in postoperative patients, especially if tamponade develops with subacute symptoms. Hypothesizing that delayed tamponade after open heart surgery is not sufficiently detected by transthoracic echocardiography, in this study CT scans were used as standard reference and were compared with transthoracic echocardiography imaging in patients with suspected cardiac tamponade.

**Method:**

Twenty-five patients after open heart surgery were enrolled in this analysis. In case of suspected cardiac tamponade patients underwent both echocardiography and CT imaging. Using CT as standard of reference sensitivity, specificity, positive and negative predictive values of ultrasound imaging in detecting pericardial effusion/hematoma were analyzed. Clinical appearance of tamponade, need for re-intervention as well as patient outcome were monitored.

**Results:**

In 12 cases (44%) tamponade necessitated surgical re-intervention. Most common symptoms were deterioration of hemodynamic status and dyspnea. Sensitivity, specificity, positive and negative predictive values of echocardiography were 75%, 64%, 75%, and 64% for detecting pericardial effusion, and 33%, 83%, 50, and 71% for pericardial hematoma, respectively. In-hospital mortality of the re-intervention group was 50%.

**Conclusion:**

Diagnostic accuracy of transthoracic echocardiography is limited in patients after open heart surgery. Suplemental CT imaging provides rapid diagnostic reliability in patients with delayed cardiac tamponade.

## Background

Pericardial effusion is common in patients after open heart surgery due to postoperative bleeding or postcardiotomy syndrome [1]. Hemodynamic relevant effusion following cardiac surgery leading to tamponade is a potentially life-treatening condition, therefore rapid diagnosis and therapy is essential [2]. Echocardiography is the standard tool to fix diagnosis and assess compromised atrial and ventricular (un)loading. Featuring excellent sensitivity and specifity in patients without previous open heart surgery, postoperative imaging with transthoracic echocardiography (TTE) is affected by modified anatomy and other issues, i.e. mechanical ventilation. Correspondingly, detection of pericardial effusion in postoperative patients can be less sensitive, and transoesophageal assessment is required [3]. Transoesophageal echocardiography (TEE) is fast and easy available in intensive care settings with ventilated patients after cardiac surgery, but represents a semi-invasive diagnostic tool, uncomfortable for spontanous breathing and not-sedated patients. Even if TEE has a high safety profile with low complication rates, an alternative diagnostic tool such as computer tomography (CT) imaging is a valuable option in patients not sufficiently assessed by TTE [4].

Hypothesizing that delayed and subacute cardiac tamponade is not detected sufficiently by transthoracic echocardiography in patients after open heart surgery, we evaluated patients with suspected non-acute pericardial effusion in regard to hemodynamic tamponade and need for intervention. Diagnostic value of transthoracic echocardiography was compared to thoracic CT scans in patients with suspected pericardial effusion postoperatively. Symptoms leading to cardiac tamponade as well as outcome of patients undergoing re-intervention are reported.

## Method

### Patients

From October 2011 until November 2012, all patients undergoing transthoracic echocardiography and thoracic CT scans due to suspected pericardial effusion after open heart surgery were included in this study. Written informed consent for diagnostic imaging was obtained, but requirement of individual patient consent was waived due to the retrospective design of the study in accordance with rules of the institutional ethical committee of University Regensburg. Patients with acute cardiac tamponade requiring immediate resternotomy in the operating room or on intensive care unit were not included in this analysis. Twenty five patients (mean 67 ± 11 years, range 50–85 years) were enrolled retrospectively. Baseline characteristics and initial cardiac surgery are shown in Table 1.

**Table 1:** Baseline characteristics of patients enrolled

	**N**	**%**
Patients	25	100
Gender		
Male	19	76
Female	6	24
Median age	67 ± 11 years	
	(50–85 years)	
Median postoperative day	21 ± 42 days	
	(1–209 days)	
Previous cardiac surgery		
CABG	n = 15	60
AVR	n = 8	32
MVR	n = 3	12
Ascending aortic	n = 3	12
Replacement		
Other (HTX, VAD, pericardectomy)	n = 3	12
Combined	n = 6	24
Redo surgery	n = 2	8

Intraoperatively, pericardium was adapted by two single sutures in case of routine cardiac surgery procedures (coronary artery bypass grafting, aortic/mitral valve replacement/repair). Pericardium was not adapted after heart transplantation, ventricular assist device implantation, aortic and redo-surgery. Two chest tubes were placed in the pericardium with additional tubes for pleural drainage, if necessary. With drainage volume less than 400 mL chest tubes were removed on first postoperative day, otherwise tubes were kept in pericardial space until drain volume felt below 200 mL per 24 hours.

Diagnostic imaging was initiated in case of new-onset symptoms indicating pericardial effusion or tamponade, respectively: collapse, syncope, dyspnea, deterioration of circulatory status (i.e. new-onset/increasing need for catecholamine support, increased central venous pressure) without other underlying pathology evident.

Patients underwent surgical re-intervention when pericardial effusion/tamponade were considered hemodynamically relevant. Hemodynamic relevance was determined as compromised (un) loading of left/right atrium/ventricle evident and correlating with clinical symptoms (see above). Suspected pericardial tamponade was confirmed, if more than 300 mL pericardial fluid/ thrombus formation were removed as well as hemodynamic status immediately stabilized (decreasing catecholamine need and central venous pressure, increasing mean arterial pressure) following pericardial relieving manoeuvre (pericardiocentesis, resternotomy).

### Echocardiography

Transthoracic echocardiography was performed with a *Philips CX50 CompactXtreme or a Philips iE33 xMatrix echo system (Royal Philips Electronics N.V., Amsterdam, Netherlands)* by physicians or cardiac surgeons experienced with more than 1000 documented transthoracic echocardiography procedures. Examination included parasternal long and short axis, apical four/five chamber view, as well as subxiphoidal view. Maximum extent of pericardial effusion was recorded, left/right atrium and ventricle were assessed in regard to unaffected/compromised (un)loading and myocardial function/contractility. Finally, operators classified echocardiography imaging sufficient or not-sufficient for assessment.

### Computertomography

Thoracic CT scans were performed with a 16-slice CT canner (*Siemens Somatom Sensation 16, Siemens Healthcare AG, Erlangen, Germany*). Reconstruction slice thickness was 1-5 mm. In case of suspected pericardial hemorrhage a volume of 120 mL contrast medium (Omnipaque, GE Healthcare, Waukesha, WI, USA) was infused intravenously (infusion rate ~4 mL/sec). For this retrospective analysis, CT findings were assessed by a radiologist unaware of echocardiography results.

### Statistics

Statistical analysis was performed with GraphPad Prism 5.03 (GraphPad Inc., San Diego, CA, USA) In descripitive analysis continuous variables are given as mean values with standard deviation. Sensitivity, specificity, positive and negative predictive values were calculated using a Fisher’s exact test. Differences of continuous variables were compared by Student’s t-test and were considered statistically significant with probability of 0.05 or less.

## Results

Twenty-five patients were enrolled in this analysis; two patients were enrolled repeatedly due to recurring tamponade symptoms. Mean time point of diagnostic imaging for pericardial effusion was 21 ± 42 postoperative days in all cases. Five patients had been readmitted to hospital, 20 patients were screened during postoperative stay. Eleven patients were on normal ward, 14 patients were on intensive (n = 12) / intermediate (n = 2) care unit. Two patients were readmitted to intensive care due to hemodynamic and respiratory collapse, respectively.

Reasons for initiating diagnostic imaging were deterioration of hemodynamic status in 16 pts. (increasing/new-onset need for catecholamine support (14 pts.), increasing serum lactate level (1 pt.), collapse (1 pt.), low-cardiac-output (3 pts.), right-heart-failure (1 pt.)), dyspnea in 7 patients, and suspected wound infection/sternum dehiscence in 7 patients, respectively. One patient suffered from recurring collapse leading to readmission, another from new-onset acute renal failure. Finally, five patients had a concomitant hematothorax. In total, 14 patients required catecholamine therapy (new-onset need in 4 patients). Inotropic (n = 8 pts., overall: dobutamine 1.875 ± 0.177 μg/KG/min or suprarenine 0.076 ± 0.033 μg/KG/min, respectively) and vasopressor support (n = 11 pts., overall: 0.093 ± 0.1 μg/KG/min) was comparable in patients with and without subsequent intervention (p = n.s.). Sixteen patients were breathing spontaneously when undergoing CT imaging. Nine patients were on ventilation therapy, 5 patients had been intubated due to respiratory collapse prior to diagnostic imaging.

### Echocardiography vs. CT

**Figure 1:**
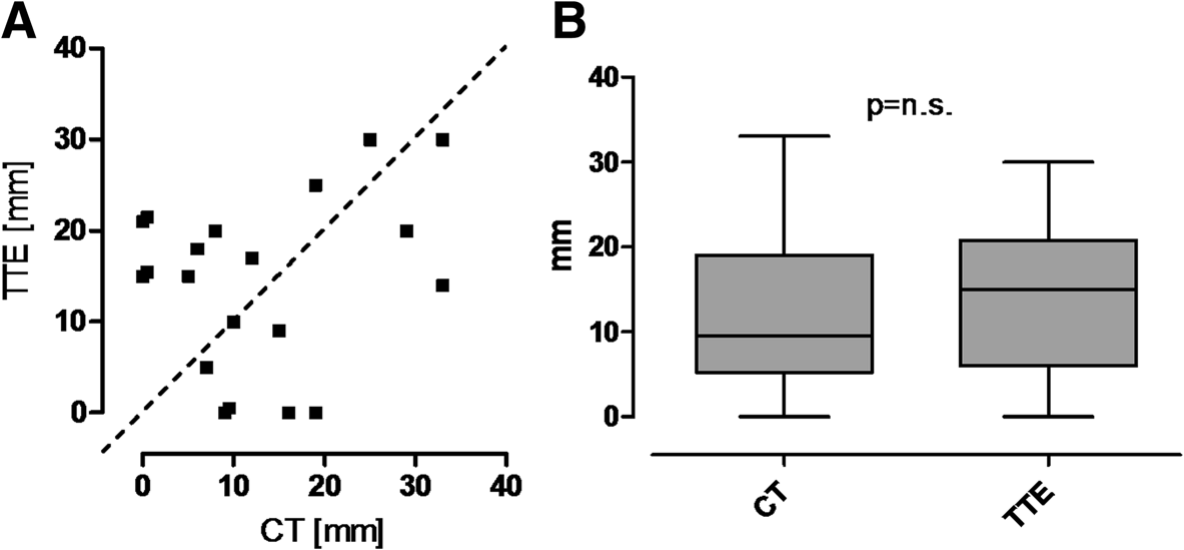
Visualization of corresponding (A) and mean (B) CT and echocardiography values of maximum pericardial effusion [mm] detected.

Transthoracic echo and CT scans matched in detecting pericardial effusion in 12 patients (44%). Mean diameters of detected pericardial effusion were equivalent (CT vs. TTE: 17 ± 11 vs. 18 ± 8 mm, p = n.s.) (Figure 1A,B). In 8 patients (30%) TTE and CT results were not consistent. Pericardial effusion was not detected positively in 4 patients by either TTE or CT, respectively. Another 7 patients (26%) did not have pericardial fluid in both screening methods consistently.

Using CT scan results as standard of reference sensitivity of echocardiography for pericardial effusion was 75%, specificity was 64%. Positive and negative predictive values were 75% and 64%, respectively.

**Figure 2:**
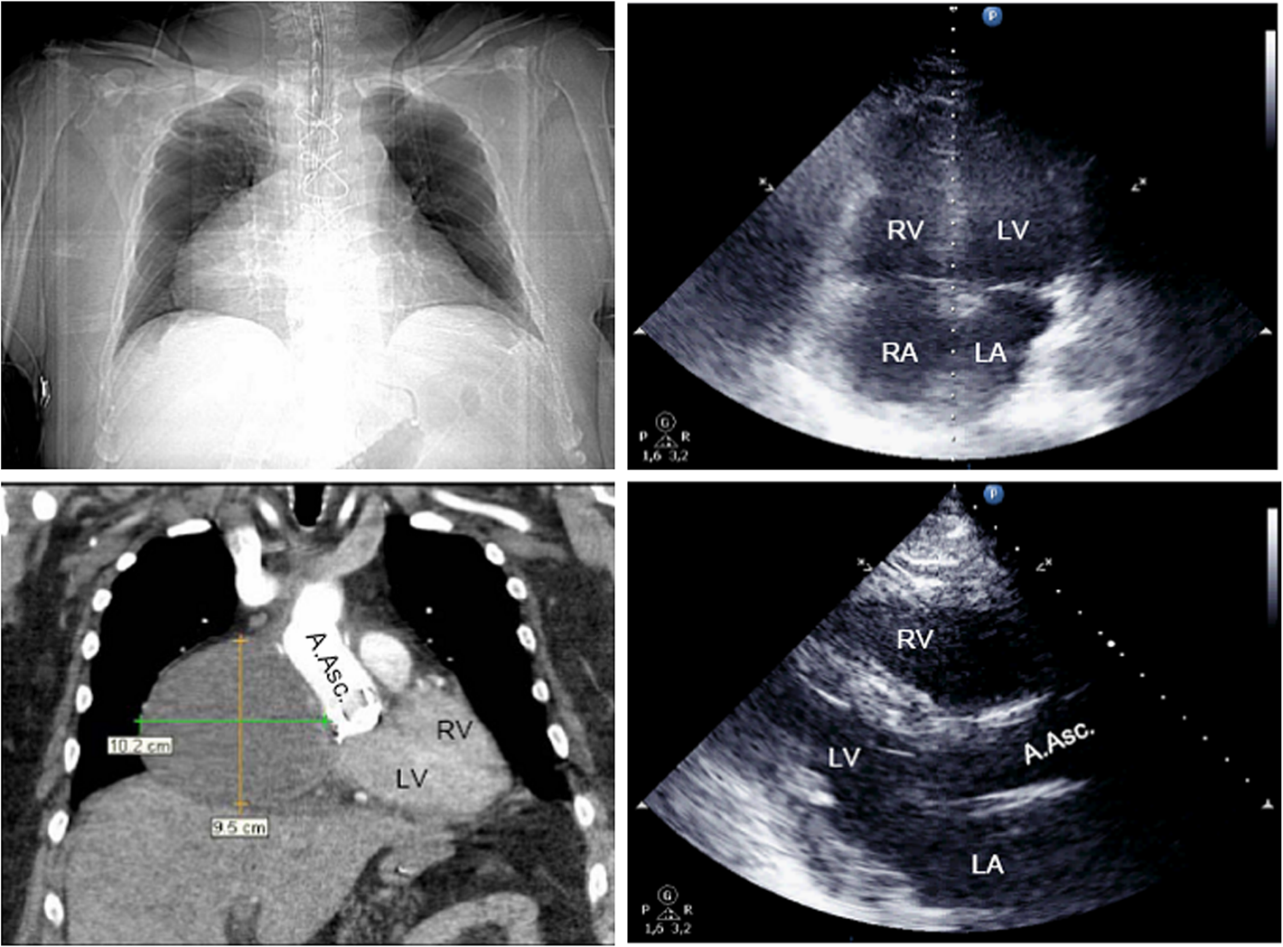
**Patient with cardiac tamponade on day 15 after ascending aortic replacement.** Right atrial hematoma (102 × 95mm) not detected by transthoracic echocardiography (B-mode: apical 4-chamber-view *right above*, parasternal long axis *right below*). Thoracic CT-imaging with contrast medium, arterial phase (topography scan *left above*, coronary view, soft tissue window *below left*). Diagnostic imaging initiated after collapse necessitating cardiopulmonary resuscitation. LV left ventricle, RV right ventricle, LA left atrium, RA right atrium, A.Asc. ascending aorta.

CT scans revealed pericardial hematoma in 9 patients (mean 54 ± 33 mm, range 19-130 mm), mainly located in the retrosternal mediastinal space (8 pts.). One patient featured a 102 × 95mm hematoma in the right atrial pericardium (Figure 2). Echocardiography sensitivity for detecting hematoma was 33% and specificity 83%. Positive and negative predictive values for hematoma were 50% and 71%, respectively (Table 2). In 7 patients, operators classified transthoracic echocardiography not sufficient to evaluate tamponade.

**Table 2:** Sensitivity, specificity, positive and negative predictive values for pericardial effusion and hematoma of transthoracic echocardiography using computer tomography scans as standard of reference

	**Pericardial effusion**	**Hematoma**
Sensitivity	75%	33%
Specificity	64%	83%
Positive predictive value	75%	50%
Negative predictive value	64%	71%

Calculation by Fisher’s exact test.

In 12 cases (~44%) pericardial effusion and hematoma were deemed as hemodynamically relevant with need for surgical re-intervention. Mean time point of re-intervention was postoperative day 20 ± 27 (range 1- 95d).

Ten patients underwent resternotomy, in 2 cases tamponade was relieved via subxiphoid pericardiotomy. Evidence for cardiac tamponade was confirmed in 11 cases. All procedures were performed without cardiopulmonary bypass support.

In fifteen cases (56%) no intervention was done, two patients received antiinflammatory medication (steroids and non-steroidal antirheumatic drugs), due to postcardiotomy syndrome. In-hospital-mortality was significantly increased in patients requiring intervention in contrast to patients without intervention (50% (6 pts.) vs. 7% (1 pt.), p = 0.009). One patient of the no-intervention group deceased after emergency CABG due to severe low-cardiac-output leading to multi-organ-failure on postoperative day 4. In the intervention group 4 patients died due to multi-organ-failure was on mean postoperative day 99 ± 115 after initial surgery. Two patients died due to respiratory and circulatory failure (on postoperative day 105 and 18), respectively.

## Discussion

Echocardiography is fundamental for imaging the heart and pericardial structures, since fast available and applicable with adequate sensitivity/specificity. Patients after open heart surgery represent a separate population with pericardial effusion up to more than 60% [1]. Effusion leading to cardiac tamponade is a potentially life-treatening condition, therefore rapid and reliable diagnostic imaging is essential in patients with tamponade suspected. Especially subacute cardiac tamponade represents a challenging entity to detect with delayed evidence of clinical symptoms. Classic symptoms, such as hypotension, tachycardia, pulsus paradoxus, increased central venous pressure, as well as low urine output can be masked after cardiac surgery or alleviated in case of slowly increasing effusion [5-7]. Of note, in this cohort interventions were done on mean postoperative day 20 with only 3 patients within 6 days after initial surgery. This indicates incidence of delayed cardiac tamponade not being affected by late removal of chest tubes. As described previously, prolonged chest tube drainage does not impact incidence of pericardial effusion [8,9]. Due to patients’ discomfort, the authors’ institutional policy implicates tube removal on postoperative day one, if drain volume is less than 400 mL without increased incidence of pericardial effusion evident.

Also, patients with and without tamponade featured comparable inotropic and vasopressor support. Therefore, prolonged/new-onset need for catecholamine support cannot be used as sensitive indicator for delayed tamponade, since unspecific hemodynamic decline is caused also by other reasons, such as infection or respiratory issues.

Moreover, majority of pericardial effusion after cardiac surgery is not circumferential but localized, then even more challenging to be detected [7]. Localized intrapericardial effusion and clots after cardiac surgery are frequently placed in the posterior pericardium or near the right atrium and ventricle with limited assessability via TTE [10]. Benefits of TTE are easy availability, high patient comfort, and comprehensive dynamic information regarding myocardial and valve function as well as intravascular volume status, but conflicting sensibility and specificity in imaging pericardial effusion have been reported [11,12]. In this analysis sensitivity and specificity were poor for both localized hematoma and circumferential effusion. Only 2 of 4 patients with pericardial hematoma requiring resternotomy were detected correctly. Ultrasound imaging is not only operator-dependent but also considerably affected by postoperative issues, such as altered anatomy, mechanical ventilation, as well as drains and trapped air in thoracic cavity, leading to not-satisfying imaging.

Correspondingly, in 7 cases TTE was classified not sufficient for assessing tamponade with additional imaging required. In face of increasing use of intrapericardial VAD systems this issue will be aggravated. In the analyzed cohort only 1 patient with LVAD with recurring tamponade was included. Diastolic collapse of right atrium and ventricle has been postulated as reliable sign for cardiac tamponade in echocardiography [13,14]. Interestingly, in patients enrolled, no diastolic collapse was detected; merely in one patient with right atrial hematoma impression of right atrium was evident. This could be caused by tamponade developing gradually without permanent diastolic collapse of the right heart. Assessability may be improved by using transoesphageal echocardiography (TEE). TEE featured high efficacy to ascertain hematoma in a recent 23 ICU patient analysis with surgically proven hematoma following cardiac surgery [15]. Even TEE is well-tolerated also in critically ill patients with few complications, our institution favours CT imaging for clarifying suspected tamponade, as it features several benefits. CT scans offer objective and not operator-dependent static imaging of thoracic anatomy. Also, other issues such as suspected sternum infection or dyspnea, or hemorrhage (evident in 56% of enrolled patients) are approached by CT scans without additional procedure.

This is particulary reasonable in hemodynamically stable patients not on ICU and at postoperative day 7 or more (in this cohort 52% and 64%, respectively) to gain detailed information about thoracic anatomy before resternotomy. Otherwise, contrast medium supported CT imaging features the risk of renal impairment and allergy-like reactions [16]. Despite this risk for imaging-related complications, a non-acute pericardial tamponade is displayed effectively via suplemental CT imaging with high sensitivity and specificity, as frequently reported after blunt chest trauma [17-19]. Therefore, CT imaging represents a valuable option to evaluate patients with suspicion of cardiac tamponade after open heart surgery.

## Conclusion

Diagnostic accuracy of transthoracic echocardiography is limited in patients after open heart surgery, since sensitivity and specificity for detection of cardiac tamponade are limited. Therefore, CT imaging is reasonable for supplemental diagnostic imaging to assess suspected delayed cardiac tamponade in high-risk patients after open heart surgery.

## Competing interest

All authors declare no conflict of interest regarding this manuscript. Also, all authors have full access and controls of data used in this manuscript, and agree to allow the journal to review, if requested.
